# Nursing Workload in Intensive Care Unit Trauma Patients: Analysis of Associated Factors

**DOI:** 10.1371/journal.pone.0112125

**Published:** 2014-11-06

**Authors:** Lilia de Souza Nogueira, Cristiane de Alencar Domingues, Renato Sérgio Poggetti, Regina Marcia Cardoso de Sousa

**Affiliations:** 1 Medical-Surgical Nursing Department, School of Nursing, University of São Paulo, São Paulo, Brazil; 2 Department of Surgery, Hospital das Clínicas, School of Medicine, University of São Paulo, São Paulo, Brazil; D'or Institute of Research and Education, Brazil

## Abstract

**Background:**

From the perspective of nurses, trauma patients in the Intensive Care Unit (ICU) demand a high degree of nursing workload due to hemodynamic instability and the severity of trauma injuries. This study aims to identify the factors related to the high nursing workload required for trauma victims admitted to the ICU.

**Methods:**

This is a prospective, cross-sectional study using descriptive and correlation analyses, conducted with 200 trauma patients admitted to an ICU in the city of São Paulo, Brazil. The nursing workload was measured using the Nursing Activities Score (NAS). The distribution of the NAS values into tertiles led to the identification of two research groups: medium/low workload and high workload. The Chi-square, Fisher's exact, Mann-Whitney and multiple logistic regression tests were utilized for the analyses.

**Findings:**

The majority of patients were male (82.0%) and suffered blunt trauma (94.5%), with traffic accidents (57.5%) and falls (31.0%) being prevalent. The mean age was 40.7 years (±18.6) and the mean NAS was 71.3% (±16.9). Patient gender, the presence of pulmonary failure, the number of injured body regions and the risk of death according to the Simplified Acute Physiology Score II were factors associated with a high degree of nursing workload in the first 24 hours following admission to the ICU.

**Conclusion:**

Workload demand was higher in male patients with physiological instability and multiple severe trauma injuries who developed pulmonary failure.

## Introduction

Many victims of trauma, due to the severity of their injuries and the need for highly complex treatment, are admitted to the Intensive Care Unit (ICU) for monitoring and continuity in the treatment of their trauma injuries. The magnitude and specificity of the care provided to these patients directly affects the nursing workload, and knowledge of this demand is essential to the management of professional staff in the critical care unit which, if appropriately allocated, will have a positive impact on the quality of care, the safety of patients with severe injuries and cost reduction [1], [2].

The concern regarding the correct allocation of nursing staff in the ICU has led to the search for a suitable tool to measure patient care services rendered during intensive care.

To this end, instruments which serve to measure nursing workload were developed in different countries, with the intention of facilitating the clinical practice of nurses by determining the actual patient demand for treatment, in terms of the size of nursing staff required and the cost analysis of the unit [3]. Among these, we highlight the Nursing Activities Score (NAS), proposed by Miranda and colleagues and validated in 99 ICUs located in 15 different countries [4]. The final score obtained by the NAS, from the analysis of 23 nursing interventions, expresses the percentage of time spent per nurse, per shift on direct patient care and in this manner contributes to the proportioning of the nursing staff in the ICU and the individual patient demand for intensive care [4].

Currently, there are no studies in the literature that analyze the results of the NAS in terms of a specific population of ICU trauma patients. However, in clinical practice many nurses are interested in this information, as it reveals the difficulty involved in caring for these patients due to the elevated workload required. Their experiences and perceptions suggest that the demand for nursing care increases proportionally according to the number of affected body regions and the location of the patient’s injuries, also depending upon the severity of the trauma and physiological instability of the patient.

In addition, it is in the interest of nurses to apply the NAS to healthcare practices, with the objective of identifying factors associated with high nursing workload in adults admitted to the intensive care unit, which may serve to assist in the management of the unit. The results of investigations have shown that demographic and clinical variables, such as length of ICU stay, mortality, severity of the patient’s condition, age, type of surgical admission and therapeutic interventions, analyzed by the Therapeutic Intervention Scoring System (TISS-28), are factors associated with the elevated workload of the nursing staff in other clinical situations [5], [6], [7].

Considering the importance of the early recognition of patients who will require a high nursing workload for the management of units caring for critically ill patients, as well as the scarcity of studies on this topic in the trauma population, this study aimed to identify the factors related to a high nursing workload in trauma victims on the first day of ICU admission.

## Methods

This is a field study, structured in a prospective cross-sectional manner, using descriptive and correlational analyses and conducted with trauma patients admitted to an ICU (specializing in this type of care) located in São Paulo, Brazil, between 2010 and 2011.

The following inclusion criteria were considered when selecting patients: being 18 years old or older; being the victim of blunt and/or penetrating trauma; and staying in the ICU for longer than 24 hours.

The project received approval from the Ethics Committee of the involved institution (*Comissão de Ética para Análise de Projetos de Pesquisa - CAPPesp da Diretoria Clínica do Hospital das Clínicas,* University of São Paulo, protocol number 1220/09) and a written consent form to participate in the study was obtained from the patients or their legal guardians.

In this study, the dependent variable analyzed was nursing workload measured by the NAS in the first 24 hours of admission to the ICU. By dividing the patients into tertiles according to NAS scores, two groups were established: medium/low workload (1^st^ and 2^nd^ tertiles) and high workload (3^rd^ tertile).

The nominal independent variables were gender, external cause of injury and origin. The region of the body with injuries that represented a potential threat to life (i.e. injuries with a score on the Abbreviated Injury Scale (AIS) ≥3) [8], the most severely injured body region and the type of organ dysfunction according to the Logistic Organ Dysfunction System (LODS) [9] were also included in this group of variables; however, in order to categorize these patients according to these parameters, we analyzed the body regions used to calculate the Injury Severity Score (ISS) [10] and the organic systems which comprise the LODS separately.

The numerical independent variables analyzed were: age; time interval between admission to the Emergency Room (ER) and admission to the ICU (in hours); the Charlson comorbidity index [11]; trauma severity according to the ISS [10] and the New Injury Severity Score (NISS) [12]; number of affected body regions (considering the six areas proposed by the ISS); number of injuries with an AIS≥3; condition of the patient according to death risk calculated by the Simplified Acute Physiology Score II (SAPS II) [13], Acute Physiology and Chronic Health Evaluation II (APACHE II) [14] and LODS, in addition to the number of compromised systems according to this index.

In this study, we investigated the behavior of different indicators of the patient’s clinical condition and trauma severity in relation to nursing workload, as we did not find this analysis in the literature in the trauma patient population.

### Statistical analysis

Multiple logistic regression was utilized to identify the factors associated with high nursing workload of patients. In the first step, a comparison between the groups (high workload *versus* low/medium workload) was performed; in relation to the nominal variables, we used the Pearson’s chi-square test and Fisher’s exact test. In the analysis of the discrete and continuous quantitative variables we used the Mann-Whitney test, since the hypothesis of normal distribution was not confirmed by the Kolmogorov-Smirnov Test.

To construct the final regression model, all independent variables which provided p<0.20 in the comparison analyses were selected and were then tested using the stepwise backward method. We used the variance inflation factor (VIF) to reach the diagnosis of multicollinearity of the final model. The predictive ability of the model was evaluated using the Receiver Operating Characteristic (ROC) Curve. In all analyses, a significance level of 5% was established.

## Results

The sample consisted of 200 patients admitted to the ICU, the majority of whom were male (82.0%). The mean age was 40.7 years (SD = 18.6) and most were patients who had suffered blunt trauma (94.5%). The most common external causes of injury were traffic accidents (57.5%), followed by falls (31.0%).

In regard to the severity of the trauma, the mean values of the ISS and of the NISS were 19.3 (SD = 9.1) and 27.1 (SD = 9.9), respectively. All of the victims had at least one AIS injury ≥3 and, on average, 2.7 (SD = 1.3) body regions were affected. A total of 65.5% of the patients had AIS injuries ≥3 in the head or neck region, and in 64% of the cases this was also one of the most severely injured body regions.

In regard to the severity of the patient’s condition, the mean and standard deviation (SD) for risk of death calculated by the SAPS II (22.9 and 22.6) and LODS (21.1 and 20.1) indexes were quite similar; however, a slightly higher result was found using the APACHE II (25.6 and 19.1). Pulmonary (76.5%) and neurological (69.0%) failures were identified in most of the victims within the first 24 hours of admission to the ICU.

Regarding nursing workload measured by the NAS in the first 24 hours of stay in the ICU, we identified that an average of 71.3 (SD = 16.9) and 69 (34.5%) patients were allocated to the third tertile and therefore formed the group that demanded a high nursing workload (NAS>75).

In Table 1, it was observed that when comparing groups (high and medium/low nursing workload), the patients who required a high nursing workload presented a higher frequency of AIS injuries scoring ≥3 in the extremities or pelvic girdle (p = 0.037). There was statistically significant difference between the groups regarding the presence of cardiac (p = 0.030), neurological (p<0.001), renal (p = 0.002) and pulmonary (p<0.001) failures, and the frequency of respective organ failure was always higher among the elements of the group with high nursing workload in comparison to the others.

**Table 1:** Comparison between the groups of high and medium/low nursing workload in relation to the nominal variables.

Variable	NAS				
	High		Medium/Low		p
	n	%	n	%	
**Gender**					
Male	61	88.4	103	78.6	0.087*
Female	8	11.6	28	21.4	0.087*
**External cause**					
Transport accident - Pedestrian or cyclist	17	24.6	23	17.6	0.327*
Transport accident - Motorcycle rider	17	24.6	38	29.0	0.327*
Transport accident - Occupant of anautomobile, truck or heavy transport vehicle	7	10.2	13	9.9	0.327*
Falls	17	24.6	45	34.3	0.327*
Other expenses	11	16.0	12	9.2	0.327*
**Origin**					
Emergency Room	15	21.7	39	29.8	0.371**
Surgical Center	51	73.9	89	67.9	0.371**
Other	03	4.4	3	2.3	0.371**
**Location of the AIS injuries ≥3**					
Head or neck (yes)	47	68.1	84	64.1	0.572*
Face (yes)	06	8.7	05	3.8	0.193**
Thorax (yes)	28	40.6	45	34.4	0.384*
Abdominal or pelvic content (yes)	11	15.9	16	12.2	0.463*
Extremities or pelvic girdle (yes)	21	30.4	23	17.6	**0.037** *
External surface (yes)	-	-	-	-	-
**Most severely injured body region**					
Head or neck (yes)	45	65.2	83	63.4	0.795*
Face (yes)	01	1.4	03	2.3	1.000**
Thorax (yes)	16	23.2	39	29.8	0.322*
Abdominal or pelvic content (yes)	09	13.0	13	9.9	0.503*
Extremities or pelvic girdle (yes)	14	20.3	16	12.2	0.128*
External surface (yes)	-	-	-	-	-
**Type of organ failure**					
Cardiology (yes)	30	43.5	37	28.2	**0.030** *
Hematologic (yes)	04	5.8	04	3.1	0.451**
Liver (yes)	05	7.2	04	3.1	0.280**
Neurological (yes)	59	85.5	79	60.3	**<0.001** *
Renal (yes)	43	62.3	51	38.9	**0.002**
Pulmonary (yes)	66	95.7	87	66.4	**<0.001** *

São Paulo, Brazil (2010–2011).

*Association test Pearson’s chi-square test,

**Fisher exact Test.

Regarding the analysis of variables that depict the severity of the trauma, we identified that the differences between the two groups occurred in relation to the ISS (p = 0.012), NISS (p = 0.003), number of affected body regions (p = 0.009) and number of AIS injuries ≥3 (p = 0.001). The values of the averages of these variables in the group requiring a high workload exceeded those found in the other group (Table 2).

**Table 2:** Comparison between groups with high and medium/low nursing workload in relation to the numeric variables.

Variables	NAS		p*
	High	Medium/Low	
	(Mean ± SD)	(Mean ± SD)	
Age	40.4±16.1	40.9±19.9	0.567
Time interval between admission tothe ER and ICU (hours)	21.0±33.2	33.8±67.3	0.235
Charlson comorbidity index	0.6±1.7	0.6±1.3	0.176
ISS	21.5±10.0	18.1±8.4	**0.012**
NISS	29.9±9.5	25.6±9.7	**0.003**
Number of affected body regions	3.1±1.3	2.5±1.3	**0.009**
Number of AIS injuries ≥3	3.6±1.8	2.8±1.8	**0.001**
APACHE II risk of death	32.8±21.4	21.7±16.6	**<0.001**
SAPS II risk of death	32.7±25.7	17.7±19.0	**<0.001**
LODS risk of death	30.0±23.4	16.4±16.4	**<0.001**
Number of compromised systems	3.0±1.0	2.0±1.2	**<0.001**

São Paulo, Brazil (2010–2011).

*Mann-Whitney Test.

It is also observed in Table 2 that there was strong statistical evidence of differences between the groups (high and medium/low workload) when comparing the number of compromised systems and the risk of death in the first 24 hours of admission to the ICU, as calculated by the APACHE II, SAPS II and LODS indexes (p<0.001). The mean values of all variables were higher in the patients who required a high nursing workload.

We identified that gender, pulmonary failure, number of affected body regions and risk of death calculated by SAPS II were factors associated with high nursing workload required by trauma patients upon admission to ICU (Table 3). In relation to the trauma severity indexes, there was prejudice in the adjustment model when the values of the ISS and NISS were tested.

**Table 3:** Logistic regression model of factors related to high nursing workload.

Variable	B	Exp (β)	95% Exp (β)	p	VIF
Gender	1.05	2.86	1.09–7.49	**0.033**	1.05
Reference: female					
Pulmonary failure	1.83	6.23	1.74–22.23	**0.005**	1.16
Reference: absence					
Number of affected body regions	0.29	1.33	1.05–1.70	**0.020**	1.01
Risk of death (SAPS II)	0.02	1.02	1.01–1.04	**0.004**	1.21

São Paulo, Brazil (2010–2011).

The odds ratio of males presenting a high nursing workload was 2.86 when compared with female patients. Patients with pulmonary failure, identified by the LODS upon ICU admission, were nearly 6 times more likely to require a high nursing workload than those without organ insufficiency. Furthermore, the addition of an affected body region, or a point in the risk of death indicated by the SAPS II score, increased the likelihood of a patient requiring a high nursing workload by 33% and 2%, respectively.

**Figure 1:**
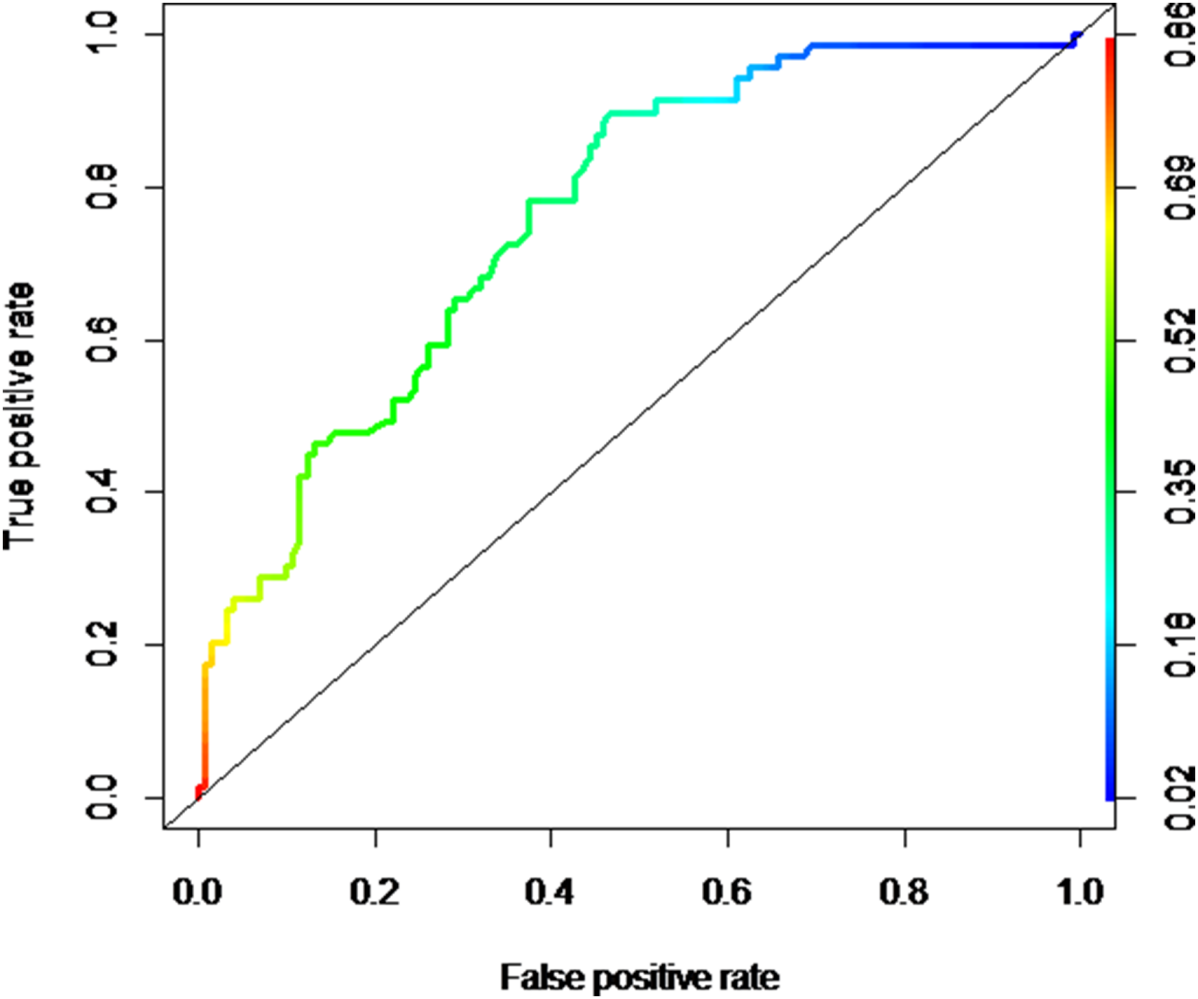
Analysis of the predictive capacity of the logistic regression model related with the high nursing workload factors. São Paulo, Brazil (2010–2011).

We did not find any indication of collinearity between the variables that remained in the final model. The area under the ROC curve was 0.763 (Figure 1), indicating a satisfactory predictive capacity of the logistic regression model for factors related to high nursing workload.

## Discussion

In this study, as in other studies that examined trauma patients in the ICU, there was a prevalence of young adult males [15], [16], [17] and patients with blunt trauma [18], [19] resulting from traffic accidents [20].

As for the severity of the trauma, the mean values of the ISS and NISS corroborate the literature [16], [18], [19], [21]. The mean values of the indexes that measure the severity of the patient’s condition (SAPS II, APACHE II and LODS) are close to those found in other studies that investigated trauma patients admitted to the ICU [15], [16], [18], [22].

The NAS average was 71.3%, a value that is higher than the value found in most studies that applied this instrument to patients admitted to general ICUs [5], [6], [23], [24], [25]. Two investigations, conducted in Brazil, addressed specific populations and found NAS means that were higher: 72.93% in the analysis of elderly patients [7] and 73.6% in a postoperative cardiac surgery unit [26]. Another study conducted in four general ICUs in Norway identified a NAS average of 96.24%. In this investigation, the authors attributed this high figure to a high demand for care related to the time spent with family and intra-hospital transport [27]. These results reinforce the idea that specialized ICUs, as well as specific internal processes, may influence the nursing workload.

In the specialist ICU that was the location of the present study, the mean NAS value showed that each patient required an average of 71.3% of a nursing professional’s working time to deliver the necessary care. The optimal ICU nurse:patient ratio was estimated to be three professionals to assist four trauma victims admitted to the critical care unit, as the mean value allows for the calculation of an NAS score of 285.2% for four patients.

Although the specific internal processes of ICUs can modify nursing staff workload, this parameter is important for nursing staff allocation in the unit, as it provides a baseline for the nurse: patient ratio and raises an alert about the higher demand that trauma victims require in relation to general ICU patients. However, in order to contribute to the daily allocation of staff according to patient care demand, it is essential to identify the individual specificities and characteristics that increase workload. In that sense, our results showed that male patients with several injured body regions, physiological instability, and pulmonary failure require more care than other trauma victims.

For decades, nursing professionals have empirically observed the relationship between severity of the patient’s condition and nursing workload in clinical practice. That perception was valued in the development and improvement of the rates that assess care needs [3], [4], [28]. In the current study, high workload was associated with the highest scores in the severity indicators of ICU patients and trauma, regardless of the measurement instrument used, showing a close relationship between the severity of the injury and the nursing workload during the first day of hospitalization in the ICU.

However, the SAPS II was the severity index that showed the best fit in the population of trauma patients considering the demand for care. This is similar to results found in studies that analyzed patients admitted to general ICUs which demonstrated that the SAPS II was a valid predictor of high nursing workload [6], [7]. Considering that the TISS-28 preceded the NAS as a tool for measuring nursing workload, it is worth mentioning that other studies show a significant positive correlation between the SAPS II and TISS-28 in analysis of ICU patients [29], [30].

Surprisingly, in this study the ISS, considered the gold standard for measuring trauma severity [31], and the NISS, developed to increase the predictive value of the ISS and facilitate its measurement [12], harmed the regression model when tested. Therefore, in our results, the indexes that described the severity of the trauma by means of victims’ primary injuries showed poorer performance than SAPS II, the index that portrayed the physiological conditions of ICU patients on the first day of ICU hospitalization. It is likely that secondary injuries, which result from primary injuries, prevailed and exerted a stronger influence on nursing workload.

This study identified that the addition of an affected body region increased the chance of the patient requiring a higher nursing workload by 33%. Considering that in this study, trauma severity was not a predictive factor for high nursing workload, we hypothesize that the increase in the number of affected body regions, together with the patient’s physiological severity, increase the time spent performing hygiene procedures, dressing changes, monitoring and titration, as well as increasing the number of nursing professionals required to move and position the patients, thus generating high NAS levels. Seguin and colleagues [16] found that the number of affected body regions influenced the occurrence of atrial fibrillation in trauma patients in the ICU, and these patients demanded a greater nursing workload as measured by the Omega scoring system.

In our investigation the presence of pulmonary failure was common among the trauma patients and had an impact on the nursing workload. In clinical practice, the nursing care provided to patients with pulmonary dysfunction is aimed at close monitoring of patients, and continuous monitoring at the bedside is often necessary for extended periods due to patient agitation, risk of accidental extubation and patient discomfort during ventilation. In addition, more professionals are needed to move and reposition patients, especially intubated patients who are sedated.

Regarding gender, research that analyzed the NAS in a cardiac ICU was similar to the results of this study, noting that the female gender was a protective factor in terms of increased nursing workload [32].

Some studies have shown that men and women exhibit different responses to trauma injuries. Male patients have more severe injuries and longer hospital stays [33], [34], as well as lower survival rates [34], in relation to women.

The impact of gender on the consequences of trauma has been discussed in various studies; animal studies have shown that being female is a protective factor following trauma and hemorrhage. For the authors, the increased testosterone or decreased estradiol and the estrogen effects on plasma cytokines result in immunosuppression in male animals and may explain the outcome advantage in female animals; however, study results do not always confirm this hypothesis [35], [36], [37].

As there was no difference between the groups (high versus medium/low workload) in relation to gender in the univariate analysis, it is inferred that the expression of gender on nursing workload is expressed in the set of conditions that are presented in the final model. What stands out is the marked severity of the patient’s condition identified by the elevated SAPS II and suggested by the presence of multiple trauma and pulmonary failure upon admission to the unit.

The satisfactory predictive capacity of the logistic regression model for the factors associated with high nursing workload reinforces the relevance of the contribution of the results of this study to clinical practice of nurses who care for trauma patients in the ICU. The application of the model to the sample correctly classified 76% of participants in medium/low workload and high workload groups (area under ROC curve = 0.763). The model, therefore, obtained a degree of accuracy that allows for recommending its use as a standard to allocate nursing professionals in the first 24 hours of care for trauma victims in ICU, when the NAS is yet to be assessed, as its calculation requires information about care for the victim within the last 24 hours in ICU.

In short, the results of this investigation strengthened the perception that trauma victims require a high workload from the nursing staff in ICU and confirmed the view that the number of affected body regions and an acute physiological instability increase the demand for care. When these characteristics were found in male individuals with pulmonary failure, a group of victims that required a high workload was early detected (NAS>75), which contributed to make an appropriate allocation of the nursing staff and ensure the quality of care with no unnecessary costs.

Despite important contributions the study has some limitations, including the small sample size and the fact that it took place in one hospital in Brazil; therefore, the results are subject to the unique characteristics of the unit and the hospital. We suggest that the results of the present study be applied with caution in clinical practice, as resources and healthcare models differ across ICU that assist trauma victims.

One should also consider that nursing workload is not based exclusively on the care required by patient, since it can also be influenced by the personal status of professional and the working environment in which he or she is inserted. These factors were not examined in this study.

## Conclusions

Male patients with physiological instability and multiple trauma injuries who developed pulmonary failure had a higher risk of requiring high nursing workload than those admitted to the ICU without these characteristics.

These results may help identify individuals requiring higher levels of nursing care, contributing to correct proportioning in terms of the size of unit staff and the allocation of team members attending to trauma patients in intensive care, in order to achieve a standard of excellence in ICU healthcare services and improve the survival and quality of life of trauma patients.
