# Correction: Nursing Workload in Intensive Care Unit Trauma Patients: Analysis of Associated Factors

**DOI:** 10.1371/journal.pone.0298922

**Published:** 2024-02-12

**Authors:** Lilia de Souza Nogueira, Cristiane de Alencar Domingues, Renato Sérgio Poggetti, Regina Marcia Cardoso de Sousa

There are errors in the Funding section. The correct Funding statement is: This study was financed in part by the Coordenação de Aperfeiçoamento de Pessoal de Nível Superior—Brasil (CAPES)—Finance Code 001. Support was provided by grant 2009/50355-4—São Paulo Research Foundation (FAPESP) to LSN. The funder had no role in study design, data collection and analysis, decision to publish, or preparation of the manuscript.
